# Reply to “Enlarged Prostate a Myth”

**Published:** 1892-09

**Authors:** H. Knapp

**Affiliations:** Lathrop, Cal.


### REPLY TO "ENLARGED PROSTATE A MYTH."

I notice in the April number (*Medical World*, page 131,) an article by J. C. Campbell, M. D., speaking rather disparagingly of the attention given to the subject of enlarged prostate, and exhorting physicians to pay more attention to "contractions and soreness of the urethra." I do not think there can be too much attention given to that most distressing and often fatal disease of the prostate gland and appendages; two fatal cases having come under my observation within a year, reminding me most forcibly of what may eventually be the means of my "taking off." It is true, that whereas I was once "young, but now am old" (nearly 80), yet never have I suffered my urethra to be impaired by the means he would have us believe. I am convinced that this affliction, diseased prostate, is much more prevalent than he would intimate, if I may j dge from the inquiries from physicians, that my article on the subject (Jan., 1891,) called forth; no less than fifteen having written of their own cases. If the people throughout the country are afflicted in the same proportion, there must be a great many suffering with it.

The "urethral contractions and soreness" he advises us to examine, are very simple ailments and easily treated, compared with a real enlarged prostate, a fact that I think he would duly appreciate if he happened to be a subject of that "*enlarged prostate rut*" he speaks of.

Now, Mr. Editor, it may appear egotistic in me to further encroach on your space or patience, in reference to my own case; but as I have already given your readers something of my experience up to Jan., 1891, I venture to obtrude still further, though the subject may be hackneyed.

In that article (Jan., 1891,) I brought saw palmetto into notice as having helped me more than anything else I ever tried, and felt very hopeful of its lasting effect, and can now say, that its action on the gland has been effective in preventing further enlargement, but it failed to relieve, except temporarily, irritation of the neck of the bladder and prostatic portion of the urethra; so I had to use the catheter occasionally.

Some four months ago, my attention was called to a new remedy called "sanmetto," composed of saw palmetto and santal. As a drowning man will "grasp at straws," so I grasped at a bottle of the remedy, and have been using it for about three months with great relief, for I have no use for the catheter now, and the deposit of mucus, instead of being an inch or more thick in a quinine bottle of urine, as formerly, is now nearly *nil*, and no pain or irritation in urinating. I think the combination of saw palmetto and santal is a happy idea—the former acting on the gland, and the latter on the mucous membrane of the bladder and urethra.

It may lose its effect as other things have, but it commends itself to my judgment as covering the pathological conditions better than any other remedy I have tried. For the benefit of those who have written me on the subject, I can say it is pleasant to take the dose, a teaspoonful about three times a day.

H. KNAPP, M. D.,
*In July Medical World.*

Lathrop, Cal.

